# Experiential learning of overnight home care by medical trainees for professional development: an exploratory study

**DOI:** 10.5116/ijme.5f01.c78f

**Published:** 2020-07-24

**Authors:** Manabu Yoshimura, Takuya Saiki, Rintaro Imafuku, Kazuhiko Fujisaki, Yasuyuki Suzuki

**Affiliations:** 1 Medical Education Development Center, Gifu University, Gifu, Japan

**Keywords:** Experiential learning, overnight home care, medical trainees, professional identity formation

## Abstract

**Objectives:**

In an ageing society, community-based medical education in a home care setting needs to be developed. Drawing on Kolb's experiential learning theory, this study aimed to explore the learning processes in overnight home care by medical trainees in terms of their understanding of terminally ill patients and their conceptualization of themselves as future physicians.

**Methods:**

An overnight home care program in which a trainee had to take care of terminally ill patients on his/her own under the supervision of a healthcare team was conducted. Nineteen trainees, including eight medical students and 11 residents, participated in this study. Text data of reflective reports written after the overnight care were collected and thematically analyzed.

**Results:**

The trainees' learning experiences in the program were categorized into four stages: predeparture, concrete experience, reflective observation and abstract conceptualization. Although they had mixed feelings, including anxiety, fear and expectations, at the predeparture stage, they could be actively involved in providing medical care and daily life support and in taking care of dying patients at the patients' homes overnight. By reflecting on their experiences, they gained a sense of achievement and identified the aspects upon which they should improve as future physicians. Subsequently, based on their reflective observation, they conceptualized their approaches to home care and the roles/responsibilities of physicians as healers, which led to professional identity formation.

**Conclusions:**

Overnight home care by medical trainees has the potential as an educational strategy to promote their realistic understanding of home care and facilitate professional identity formation.

## Introduction

The provision of effective home care for elderly and terminally ill patients is essential to a rapidly aging society. Since the percentage of people in Japan aged 65 or older is 28.5% as of 2019, it is an urgent issue.^1^^-^^2^ Given this context, community-based medical education (CBME) in a home care setting should be developed to foster the development of health professionals who can better understand the biopsychosocial (BPS) aspects of patients and their family members and provide comprehensive patient care.^3^^-^^5^ In other words, students need to understand the contexts in which people live, the ways in which home services are delivered and the complexity of home care.^6^

Home visits as an educational strategy are commonly incorporated into CBME programs. Previous studies argue that home visits to patients are a highly impactful practice in undergraduate medical education.^7^^-^^8^ Henley argues that home visit programs can afford the opportunity to introduce a broader BPS 'spiritual' approach that allows students to recognize the ethical issues and roles of religion and faith in coping with illness.^9^ Moreover, O'Donnell and colleagues investigated first-year medical students' learning experiences of attending house call practice in geriatric medicine.^10^ In their study, the students could pay more attention to the functional impairment of elderly people and realize the importance of family support for the patients. Yuen and colleague.^11^ found that students' experience in the half-day house call program led to enhanced empathy and sensitivity toward chronically ill patients. Likewise, other studies found that medical students showed greater interest in geriatric medicine and gained a better understanding of the 'reality' of elderly people's lives after a home visit nursing program.^8^^, ^^12^^-^^14^

However, in Japan, most clinical education programs for medical students and residents are provided in the hospital setting. As a result, they learn to approach clinical practice primarily from the viewpoint of inpatient care.^15^ Of the small body of home visit programs, most of them are usually provided in the daytime, when the patients can receive satisfactory care from caregivers and adequate support from their family. From an educational viewpoint, trainees or students learn in a safe environment organized mainly by their supervisors during the daytime. However, the anxiety of elderly patients and their families increases more at night. Addressing anxiety in elderly patients is an essential competency for health professionals since patients' anxiety is a significant risk factor for the progression of disability.^16^ In fact, there are many cases in which physicians and other health professionals need to rush over to the patients' homes at night and address problems of patients' physical dysfunctions, taking their psychosocial aspects into account.^17^^-^^18^ It is important for health professionals to fully understand patients' social lives and changes in their physical and psychological conditions over the course of a whole day and night to provide holistic patient care in a home visit setting.

Hughes and colleagues also suggest that homestay experiences in the host family positively affected medical students' communication, cultural competence and professional skill development.^19^ In this sense, staying at elderly patients' homes overnight can be a better opportunity for students to learn a way of providing patient care for elderly patients and their families in the community from a holistic perspective. Here, we developed and offered students/residents an innovative learning intervention called the "overnight stay at terminally ill patients' homes (OSTIPH)". This study aimed to explore the trainees' learning experiences in this newly developed program of CBME. To achieve the aim of this study, we developed the following research questions:

·       What did trainees experience, feel, and learn from the OSTIPH?; and

·       How do these experiences and lessons contribute to the formation of trainees' professional identity?

### Theoretical framework

This study adopts the experiential learning theory as the theoretical framework that provides a holistic model of the learning process.^20^ The conception of experiential learning is an established approach in the tradition of adult education theory.^21^ With an emphasis on the idea that experience plays the central role in the learning process, experiential learning theory is a theoretical synthesis of Dewey's philosophical pragmatism, Lewin's social psychology and Piaget's cognitive development. Kolb defines learning as "the process whereby knowledge is created through the transformation of experience.^20^ Knowledge results from the combination of grasping and transforming experience. Kolb proposed a model in which this transformation of experience occurs in a cyclical fashion of four stages: concrete experiences, reflective observation, abstract conceptualization, and active experimentation. According to the four-stage learning cycle, concrete experiences are the basis for observations and reflections. These reflections are assimilated and distilled into abstract concepts from which new implications for action can be drawn. These implications can be actively tested and serve as guides in creating new experiences.^22^

Moreover, at the stages of abstract conceptualization and active experimentation that leads to the next learning cycle, learners' reflective practice would affect their identity formation, particularly in professional education. Key elements of professional identity formation include experiential and reflective processes.^23^^-^^24^ In this sense, the concept of professional identity formation is key to exploring students' or residents' learning experiences in the OSTIPH. Physician's professional identity is defined as "a representation of self, achieved in stages over time, during which the characteristics, values, and norms of the medical profession are internalized, resulting in individual thinking, acting, and feeling like a physician".^25^ In this regard, Cruess, Cruess and Steinert argue that a fifth level 'is' should be added at the apex of Miller's pyramid (i.e., knows, knows how, shows how, and does) in the movement to ensure that professionalism is taught throughout the continuum of medical education.^26^ 'Is' refers to being or identity-related to "consistent attitudes, values and behaviors expected of one who has come to think, act, and feel like a physician".^27^

## Methods

### Study design

Drawing on Kolb's model of the learning cycle^20^ with the concept of professional identity formation,^26^ this study will examine the students'/residents' internal cognitive processes through their experiences of staying at the patients' homes overnight by qualitative research. Specifically, this study focused on describing the students'/residents' learning processes, including the following: 1) having an experience with elderly patients at their home overnight; 2) reflecting on the experience; 3) learning from the experience, and 4) trying out what they have learned. Furthermore, this study also assumes that social interactions with attending physicians, other health professionals, patients and family members can be the key to better understanding residents' learning processes.

### Study setting and educational intervention

The OSTIPH was introduced and developed in the Ibi Community Medical Center in central Japan in 2005.^28^ Three general practitioners work as full-time medical staff in this center and mainly engage in home care in Ibi District in Gifu Prefecture. They offer a short clinical training program of CBME (for two to four weeks) for medical students and residents. Approximately 50 trainees participate in this program every year.^29^

The main objective of the OSTIPH program is for trainees to learn a whole process of providing daily life support and healthcare support for patients and their families in a home visit setting by staying overnight with them. Specifically, the trainees in the OSTIPH are expected to fully understand the life circumstances of the patients and explore the problems of continuing home care through the experience of both providing patient support by themselves and observing how health professionals in a team carry out patient support and caregiving. They are also encouraged to reflect on what they have learned through experience in this program every day.

At the beginning of clinical training at the Ibi Community Medical Center, each trainee was assigned an elderly and terminally ill patient with the "do not attempt resuscitation (DNAR)" status who was provided home care. When the trust relationship was established, the OSTIPH was implemented. The OSTIPH is a 2-day program. On day 1, before the home visit, the supervisors give an induction session to explain the aims of the OSTIPH to the trainees, particularly with an emphasis on the fact that the patient has a DNAR status. The patients and their family members have also received information about the OSTIPH and the roles of the trainees from the supervisors in advance. The trainee and healthcare team members visit the patient's home in the afternoon. The trainee is required to bring healthcare supplies and his/her bedclothes for an overnight stay. In the evening, the healthcare team members return to the center, and only the trainee remains at the patient's home. The trainee is expected to communicate with the patient and family and provide healthcare support for their daily activities overnight. In case the patient is in critical condition at night, the trainee must call a healthcare team member immediately. In such cases, the supervisor would rush to the patient's home and handle his/her problems. On day 2, the trainee returns to the center from the patient's home at 8 am. Then, a debriefing/feedback session is held among the trainee and the healthcare team members. The trainee is required to report what he/she experienced and learned in the OSTIPH based on the information from medical records and his/her own reflections.

### Research participants and patients

This study selected 19 research participants, including 8 fifth- or sixth-year medical students (all males, code "a" to "h") and 11 PGY1 and PGY2 residents (1 female and 10 males, code "i" to "s") who were all Japanese and from medical schools and teaching hospitals from all over Japan and joined the clinical training program of CBME offered by the Ibi Community Medical Center during the period from April 2014 to March 2015. All but several female trainees who cared for male patients were asked to join the OSTIPH, and all but one trainee agreed to participate.

Sixteen patients who received home care from the medical center agreed to cooperate on the OSTIPH (see Table 1). All of them were Japanese, 11 patients were over 80 years old, 12 were male, and 4 were female. Eleven patients were terminally ill and agreed to DNAR status. Three patients died during the OSTIPH, and 8 patients died within a week after the OSTIPH. Thirteen patients were cared for by a family member, and three were living alone.

**Table 1:** Demographic information of the patients

**Case**	**Age**	**Gender**	**Disease**	**Stage**	**Death**	**Caregiver**	**Attending** **participants**
**1**	80s	F	Heart failure			-	**k**
**2**	70s	M	Malignant lymphoma	Terminal	^‡^	Wife	**e**
**3**	80s	M	Gastric cancer	Terminal	^†^	Daughter	**l**
**4**	80s	F	Dementia, heart failure	Terminal	^‡^	Husband	**f**
**5**	80s	M	Stroke			Wife	**m**
**6**	80s	M	Renal failure, uremia	Terminal	^‡^	-	**g**
**7**	40s	M	Alzheimer’s dementia			Parents	**n**
**8**	80s	F	Heart failure, dementia			Husband	**a**
**9**	90s	M	Renal failure, uremia	Terminal	^‡^	Daughter	**b**
**10**	80s	M	Gastric cancer	Terminal	^†^	Son	**o**
**11**	80s	M	Laryngeal cancer	Terminal	^‡^	Daughter	**i, j, p, c**
**12**	60s	M	Pancreatic cancer	Terminal	^‡^	Wife	**q**
**13**	70s	M	Gastric cancer			-	**d**
**14**	80s	M	Heart failure	Terminal	^‡^	Son	**r**
**15**	70s	M	Leukemia	Terminal	^‡^	Wife	**s**
**16**	80s	F	Pancreatic cancer	Terminal	^†^	Husband	**h**

All the patients were Japanese. ^†^Death during overnight stay. ^‡^Death within 7 days after the program

The Institutional Review Board approved this study at Gifu University. The principles of the World Medical Association and the Declaration of Helsinki were applied to this study. Written informed consent was obtained from all participants of this study. All participants were informed that the participation is voluntary, their comments would be kept anonymous, and their quotations would be included in the paper.

All the patients were Japanese. †Death during overnight stay. ‡Death within 7 days after the program

### Data collection and analysis

This study collected data from the trainees' reflective reports, in which their experiences, feelings and thoughts during the OSTIPH were written immediately after their overnight stay at the patient's home. The structure of this reflective report was developed based on Kolb's ELT.^20^ In other words, the trainees were asked to write about their actual experiences during the OSTIPH (concrete experiences), their feelings and acquired knowledge after the experiences (reflections), the impact of their experience on their own personal and professional development (abstract conceptualization), and their thoughts on their future careers related to their experience in the OSTIPH (active experimentation) (see Table 2).

**Table 2:** Reflective learning report

1.	Describe the daily living and caregiving activities you experienced at the patient's home. (concrete experience)
2.	How did you feel when you were invited to participate in the overnight homestay experience? (concrete experience, reflection)
3.	Describe the emotions you felt and the lessons you learned. (concrete experience, reflection, abstract conceptualization)
4.	Describe the impacts of the overnight stay at the patient's home on your personal/professional growth and identity formation. (reflection, abstract conceptualization, active experimentation)

Drawing on 'the stages of experiential learning' as classified by Kolb, the trainees' perceptions of learning in the OSTIPH and thoughts about their future careers were examined with a more inductive labeling process for qualitative data. More specifically, a thematic analysis approach was employed to inductively generate and develop salient categories of their experiences.^30^ To enhance the trustworthiness of the qualitative analysis, two researchers (MY and YS) were independently involved in coding and categorizing the data before cross-checking their data interpretation and analysis. The preliminary results were carefully reviewed multiple times by all the members of the research team.

## Results

**Figure 1:**
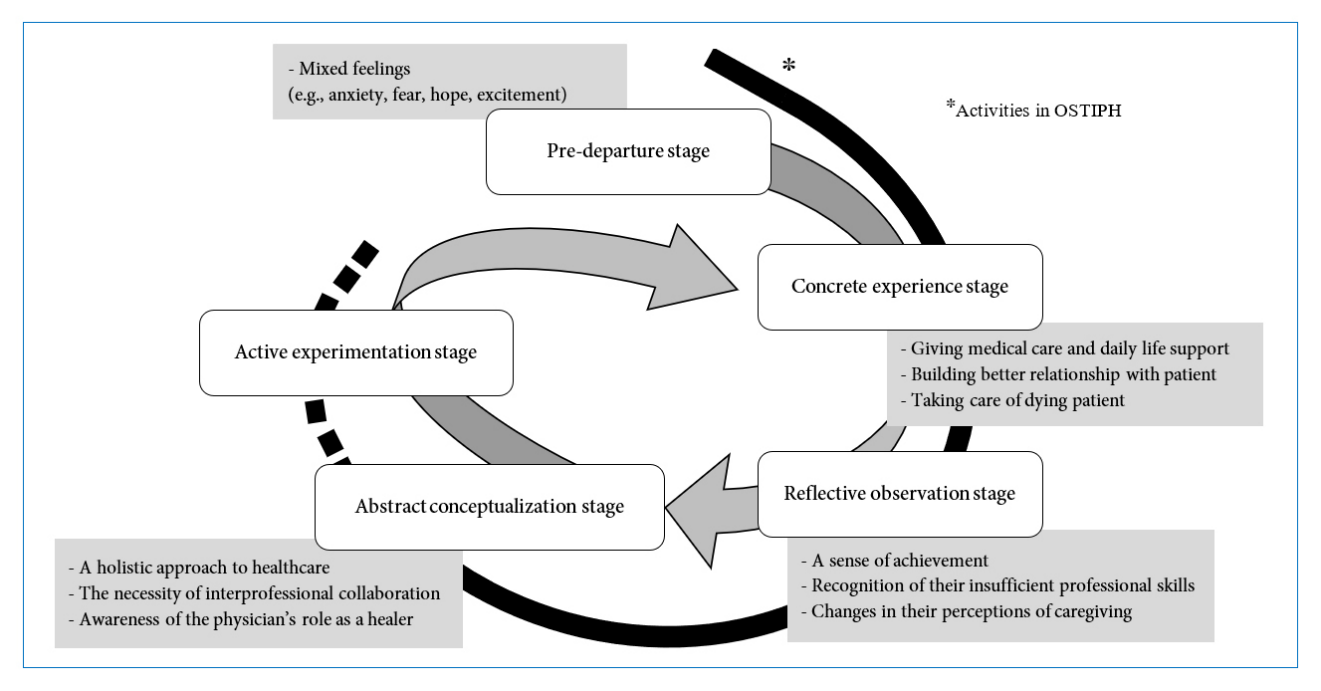
Overview of the trainees' activities in the program

In this study, the trainees' learning experiences in the OSTIPH were categorized into the following four stages: predeparture to the program, concrete experience, reflective observation and abstract conceptualization stages (see Figure 1). It should be noted that the trainees hardly described their activities as being at the active experimentation stage. In the following sections, the details of their activities at each stage will be presented.

### Predeparture stage

The trainees had mixed feelings about their participation in the OSTIPH at the predeparture to the program; these feelings included hope, excitement, anxiety, fear, and nervousness. Some trainees had positive affect toward staying at the patient's home. For example, trainee (n) said,

"I had felt excited because I would be given an opportunity to see the true life of the patient and family."

In other words, the trainees aimed to better understand the complexities of the activities and discourse of the patient and family in the given context. Moreover, they already had a feeling of gratitude toward the patient and family at this stage. For example, trainee (f) said,

"I was grateful that the patients and families were willing to accept me as a participant in this program. To requite their kindness, I would work hard for them."

On the other hand, most trainees had stronger negative emotional states than positive affect, which were induced by their lower confidence in their professional skills to handle uncertain and unexpected patient situations and to provide satisfactory care for the family. For example, trainee (o) said,

"I heard that as the condition (of the patient) was not good, the patient would possibly pass away during my stay at the patient's home. When I knew about this situation, I was worried about whether I could handle this on my own. And, I was worried that the family members would be upset if this should happen, since only I, a trainee, was with them."

Although they had complicated feelings, all the trainees prepared themselves before starting the program for some challenging situations that would happen during their stay at the patient's home. Trainee (g) said, "I was told what would happen in this program by a colleague before. So, the only health professional at their home would be me. Even in a serious situation, I would have to work for them as a (future) doctor."

### Concrete experience stage

The trainees gained a variety of experiences through interaction with the patient and his/her family members overnight. First, the trainees provided support for the daily life of the patients and their family. In particular, spending time with the patients and families themselves, such as by cooking, dining, watching TV and taking their dog for a walk together, facilitated the trainees' understanding of the lifestyle of the patients and families situated in the social context. Moreover, the trainees could learn through casual conversation with the family members about their experiences and true feelings regarding caregiving at home. For example, trainee (i) said,

"I talked over coffee at night with patient's daughter. She stated that caring for the patient was harder and more stressful than she thought. I could understand the importance of short stays to reduce the burden of care."

As they communicated more with each other and were involved in supporting the patient, the trainees strongly felt that they could build better relationships with the patient's family. They found that the family members welcomed them and relied on them as health professionals. For instance, trainee (p) said,

"His wife was very pleased when I was assigned to stay at her home, so I was thinking about the best approach. At the beginning, the patient apologized and hesitated to state his request. But during the night, he gradually told me that he felt so anxious and nervous. This was so impressive for me."

Second, as the trainees were the only health professionals at the patients' homes, they were expected to provide basic healthcare support, such as vital sign measurement, the suctioning of sputum, toileting assistance, meal assistance, assistance with turning over in bed and night calls. Trainee (i) said,

"I did the suctioning of sputum through the tracheal tube every hour by using the tube."

Some trainees experienced a more serious situation, as trainee (l) commented,

"When I was checking the patient's respiratory condition, I noted that it was getting worse. So, I called the center urgently urgently, and after the supervisor came, we worked hard to care for the patient, who eventually passed away."

Out of 19 trainees, six experienced taking care of a dying patient during their stay at the patient's home or knew that the patient had died soon after the trainee returned to the center. This experience appeared to be an influential event in their careers as doctors. For instance, trainee (d) said the following:

"The patient I was taking care of died and stopped moving. It was an unforgettable and shocking event. I was surprised that the patient's daughter seemed to be relieved, but I think it is understandable."

Furthermore, it was an opportunity to consider how the trainees had to behave by taking the family members' feelings and attitudes toward the reality of the patient's death into account. For example, trainee (s) could directly discern a family member's feelings about the patient's death:

"When I visited the patient's home the next morning, I confirmed his death. His wife stayed calm and said to the patient, 'Your doctor is here to visit you. He kindly cared for you last night.' I felt that her words to the patient were actually a form of positive feedback on my involvement in his death."

### Reflective observation stage

The trainees reflected on their experiences of direct interaction with the patients and their family in the OSTIPH in terms of their own behavior, feelings and views of home care. Although they had strong anxiety about their participation in this program at the predeparture stage, their negative affect was gradually removed through their active involvement in patient support. At the same time, they started to feel a sense of achievement to some extent and began to regard the OSTIPH as a positive learning experience. For example, positive feedback from the patients and families led to the trainees' confidence in their professional skills. Trainee (d) said,

"I was very pleased to hear that the patient was comfortable with the posture change I did. Although I was anxious about how to do it, the patient's comment made me feel rewarded."

On the other hand, at the reflective observation stage, they realized that they could not do anything on their own and needed help from more experienced people. Specifically, they identified that there were many points to be improved, including observational, procedural and decision-making skills. For the trainees who had not had enough clinical experience, it was difficult to address patients' problems in real-life situations on their own. For instance, trainee (e) said the following:

"I was truly worried if I would overlook any critical changes in the patient's condition. If this happened, I couldn't have judged if it would be the onset of a serious problem. Should I call my teacher? I didn't know when to call him. I regretted my lack of knowledge and smaller amount of clinical experience."

In addition to reflecting on their professional skills, they recognized that as they had not directly observed patients' real lives in a social context, they had not seen the patients from various aspects. Particularly, they realized that they only looked at one side of the patient when they were hospitalized. For example, when dining with the patient, trainee (k) found that the patient could do many housework tasks by himself, which was not seen in the hospital. Trainee (k) said,

"The patient was able to put the soup in a bowl with a ladle without spilling it. When the patient was in the hospital, I had never imagined he could do housework like this after discharge."

Moreover, they reflected their own perspectives on caregiving through their observation of the behavior and psychological state of family members as caregivers. Specifically, the trainees noticed that they focused exclusively on a negative aspect of caregiving, such as troublesome, time-consuming and tough tasks. However, in talking about experiences and beliefs regarding caregiving with both the patients and family members, the trainees found there was also a positive aspect of caregiving. Their reflection on the discussions of caregiving with the 'stakeholders' allowed them to have a new perspective on caregiving and healthcare in the community. For example, trainee (m) said,

"His wife seemed to feel at ease that the patient was at home. I felt that sometimes caregivers themselves could be saved by caregiving."

Furthermore, trainee (b) said,

"The daughter-in-law and grandchildren all seemed to enjoy caring for the patients. I was grateful that they had accepted me. I have never been so worried about another person as I was this time."

Finally, trainee (p) remarked,

"The family said it was very nice that the family could say goodbye with their hands before the patient was gone. I was lucky enough to be there for this last moment."

### Abstract conceptualization stage

The trainees conceptualized the approaches to healthcare and the roles/responsibilities of medical doctors through reflective observation on their concrete experience in the OSTIPH. The abstract conceptualization in their mind can be connected to their professional identity formation because they could gain a more holistic viewpoint toward healthcare and a new perspective on their future careers as physicians. Specifically, they realized the importance of understanding the psychosocial aspects of the patient to provide patient-centered medical care and that the physician should take the role of a healer who respects for the autonomy of the patients as a member of the healthcare team. For instance, trainee (m) said about a holistic approach to patient care,

"I was able to obtain insights into the patient- and family-centred medical care, which will be my lifelong goal for achievement in my career."

Moreover, trainee (h) said the following:

"I want to become a doctor who can propose the best care plan for home visits by taking the patient's desires into account. For example, for a patient who is unable to recover his/her disease completely and who does not want any further treatments at the hospital, health professionals have to preferentially ensure the patient's quality of life."

Regarding the importance of understanding the patients' psychosocial aspects, trainee (b) said,

"From now on, I try to pay more attention to not only patients' physical state but also their mental state, their everyday life situations, and their human relations."

In relation to the provision of comprehensive patient care, they also recognized the necessity of interprofessional collaboration for community-based medicine through their experience in remaining alone at the patient's home as a 'physician.' In the program, they found that what could be offered only by the physician was relatively limited. Trainee (f) said,

"I found that collaboration with other professionals is essential to the successful provision of home care, because I truly understood that better medical service could not be offered only by the doctor."

## Discussion

This study has drawn on Kolb's experiential learning theory ^20^ to explore trainees' learning experiences in the OSTIPH. This study found that they experienced providing medical care and daily life support, and some of them had mixed feelings about taking care of dying patients. As they were building better relationships with the patients and families, they gradually felt a sense of achievement toward their participation. At the same time, through interaction with the patients and families, they could clarify the aspects they should improve upon to become an 'independent' doctor. Their reflective observation of concrete experiences led to their abstract conceptualization of holistic approaches to healthcare and the roles/responsibilities of physicians. Therefore, although the OSTIPH is only a 2-day program, it is an influential learning opportunity in which trainees could deeply think about what healthcare and physicians should be.

One of the key features of this innovative program is related to the learning environment. The trainees must remain engaged in patient care as a novice health professional at the patient's home on their own after the medical team returns to the center. In other words, by creating an environment in which they are expected to assume responsibility for patient care, the trainees' ownership of learning in the OSTIPH has been enhanced effectively. One way to improve student achievement is through supporting student ownership of learning.^31^ Through 'doing' on their own in this program, the trainees perceived that they had gained benefits, such as the development of clinical and communication skills, as well as broadened their perspectives on healthcare and identity formation. In other words, a lack of autonomy led to disengagement and a lack of ownership over patient care.^32^

However, as the trainees described their feelings at the predeparture stage, this context of learning can be emotionally stressful, although we made careful efforts to establish trusting relationships between the trainees and families before the OSTIPH, as described in the study setting. In particular, it is natural that they felt anxious and fearful in taking care of terminally ill patients at potential risk of death. This finding in this study is congruent with that of a study by Arai et al,^33^ who investigated the emotions of residents taking care of dying patients in general hospitals. They argue that the residents' emotional state was negatively affected by not only the reality of patient death but also their relationship with their supervisors who were biomedically oriented. In this situation, the supervisors would be expected to provide feedback for the trainees in accordance with the BPS model of patient care. Although a certain level of environmental stress positively influences learners' performance,^25^^-^^26^ we must ensure a 'safe' environment by monitoring the emotions of trainees and patients, such as holding an elaborate induction session to have participants understand the objectives of the

program, providing better accessibility to medical team backup during the stay, and a holding follow-up session after their overnight stay. It is also important for supervisors to establish better social relationships with patients so that the patients and families feel free to contact the doctor during the trainee's stay if necessary.

In the OSTIPH, the trainees could gain a better understanding of the patients' lifestyle and changes in their emotional and physical conditions overnight. The findings of this study are congruent with those of previous studies examining the changes in students' experiences in home care training.^14^^-^^15^ The trainees in the OSTIPH were encouraged to take an ethnographic approach that focuses on "an in-depth description and understanding of cultural patterns within the particular contexts" among patients and their family members.^34^ Spending time with the patients and families themselves, such as through cooking, dining, watching TV, and taking a walk, is essential to a comprehensive understanding of patients from the BPS viewpoint. Previous studies argue that home visit experiences allow students to better understand the functional impairment of patients and ways of offering support to the family in a particular context.^10^^-^^11^ However, almost all home visit programs have been conducted in the daytime, during which the students cannot learn how patients feel and what caregiving activities their families are performing at home in the nighttime. In this sense, the OSTIPH added a new component to existing educational practices in terms of facilitating students' understanding of the usual 24-hour lives of the patients and families at home or in a given community.

Reflective observation of concrete experiences in the OSTIPH resulted in changes in trainees' perceptions of healthcare and physicians' roles/responsibilities in interprofessional teams at the abstract conceptualization stage. This conceptualization of oneself as a future physician in healthcare through experience in the OSTIPH would become the foundation of their professional identity formation.^23^ Critical events shape identities, which involve self-examination with feelings of guilt or shame as an initial factor of transformative learning.^35^ In other words, emotional experiences in the OSTIPH, such as taking care of dying patients at night, would be a turning point in their professional identity formation. Regarding the relationship between emotions and identity formation, Cruess et al^36^ argue that although novice trainees may be overwhelmed by their emotions at the beginning of the event, they can better control their emotion as they obtain a better understanding of their own professional roles. Therefore, to facilitate their professional identity formation, the supervisors need to change the degree of providing instruction to each trainee depending on their emotional state at the predeparture stage.

Trainees' professional identities were developed through the establishment of better relationships with the patients and families.^37^ For example, the sudden loss of a patient, which was a shocking and unexpected event for the trainees, led to their deeper reflection on what the physician could have done for the patient. Moreover, when family members expressed their gratitude for the trainee's support after a patient's death, they realized that physicians should take roles of not only curer but also healer. Specifically, in the reflective report, the trainees emphasized the importance of having respect for the patient's will, giving reassurance to family, and being considerate of the patient's feelings, which are considered the attributes of healers.^26^^, ^^38^

At the abstract conceptualization stage, the trainees could develop their professional identity by gaining new perspectives on healthcare and the roles of physicians in a home care setting. However, in their reflective report, most trainees in this study did not describe their activities at the active experimentation stage. Active experimentation can serve as the stage for strengthening trainees' professional identities through testing their conceptualization of healthcare and oneself as a future physician. This is partly because the OSTIPH is a single short-term intervention, and there might be less opportunity for carrying out active experimentation. To facilitate their active experimentation for gaining new experiences, the trainees are ideally encouraged to participate in the OSTIPH several times. As Cruess et al^27^ and Javis-Selinger et al^39^ suggested, promoting learners' professional identity formation is closely connected with the development of competency as a future physician in the community. Therefore, CBME, such as the OSTIPH, needs to be further developed and expanded.

## Conclusions

This study found that an overnight stay at a terminally ill patient's home could afford a rich opportunity for trainees to better understand the context in which patients and families live and to learn about ways of delivering home care. Moreover, their reflection on these learning experiences led to active conceptualization of approaches to healthcare, which resulted in the development of their professional identities.

The results were not generalizable due to the relatively small number of participants and rather short-term implementation in a specific medical center. Moreover, the cultural and social aspects unique to Japan that may have affected the trainees' experiences in home care were less examined in this study. However, this study fully described the trainees' learning experiences during their overnight stay at terminally ill patients' homes and suggests that this innovative program in CBME can be applied internationally to the contexts of other institutions and communities. The findings of this study corroborate the existing body of studies emphasizing the incorporation of home care as an educational activity in medical education.

For further research, a follow-up investigation is necessary to clarify the long-term impact of this program in terms of professional identity formation. Moreover, it is worthwhile to perform semi-structured interviews and participant observation to conduct a more in-depth exploration of the experiences of not only the trainees but also the patients and their families in the OSTIPH.

### Acknowledgments

The authors thank the healthcare staff who practiced home visits, the patients and families who cooperated, and the trainees who participated in this study. We also express our deep gratitude to Mr. Phillip Evans, University of Glasgow, and Prof. Erik Driessen, Maastricht University, for their helpful advice and encouragement.

### Conflict of Interests

The authors declare that they have no conflict of interest.
